# Novel selective β_1_-adrenoceptor antagonists for concomitant cardiovascular and respiratory disease

**DOI:** 10.1096/fj.201601305R

**Published:** 2017-04-11

**Authors:** Jillian G. Baker, Sheila M. Gardiner, Jeanette Woolard, Christophe Fromont, Gopal P. Jadhav, Shailesh N. Mistry, Kevin S. J. Thompson, Barrie Kellam, Stephen J. Hill, Peter M. Fischer

**Affiliations:** * Cell Signalling Research Group, School of Life Sciences, University of Nottingham, Nottingham, United Kingdom;; † School of Pharmacy, University of Nottingham, Nottingham, United Kingdom;; ‡ Centre for Biomolecular Sciences, University of Nottingham, Nottingham, United Kingdom

**Keywords:** β-blocker, selectivity, heart disease, asthma

## Abstract

β-Blockers reduce mortality and improve symptoms in people with heart disease; however, current clinically available β-blockers have poor selectivity for the cardiac β_1_-adrenoceptor (AR) over the lung β_2_-AR. Unwanted β_2_-blockade risks causing life-threatening bronchospasm and reduced efficacy of β_2_-agonist emergency rescue therapy. Thus, current life-prolonging β-blockers are contraindicated in patients with both heart disease and asthma. Here, we describe NDD-713 and -825, novel highly β_1_-selective neutral antagonists with good pharmaceutical properties that can potentially overcome this limitation. Radioligand binding studies and functional assays that use human receptors expressed in Chinese hamster ovary cells demonstrate that NDD-713 and -825 have nanomolar β_1_-AR affinity >500-fold β_1_-AR *vs*. β_2_-AR selectivity and no agonism. Studies in conscious rats demonstrate that these antagonists are orally bioavailable and cause pronounced β_1_-mediated reduction of heart rate while showing no effect on β_2_-mediated hindquarters vasodilatation. These compounds also have good disposition properties and show no adverse toxicologic effects. They potentially offer a truly cardioselective β-blocker therapy for the large number of patients with heart and respiratory or peripheral vascular comorbidities.—Baker, J. G., Gardiner, S. M., Woolard, J., Fromont, C., Jadhav, G. P., Mistry, S. N., Thompson, K. S. J., Kellam, B., Hill, S. J., Fischer, P. M. Novel selective β_1_-adrenoceptor antagonists for concomitant cardiovascular and respiratory disease.

β-Adrenoceptor (AR) antagonists (β-blockers) block catecholamines from binding to β-AR, which reduces heart rate (HR) and the force of contraction, thereby reducing myocardial oxygen demand (1, 2). Randomized, placebo-controlled clinical trials demonstrate mortality reductions with β-blockers of 34 to 35% in patients with heart failure and of 36 to 39% in patients with myocardial infarction (3–7). β-Blockers are therefore recommended for all patients with heart failure or a recent myocardial infarction, as well as being first-line therapy for atrial arrhythmias and having important roles in hypertension, thyrotoxicosis, portal hypertension, migraine, glaucoma, and anxiety.

Bisoprolol (8) is one of the most β_1_-selective β-blockers clinically available and, although often called cardioselective, has poor β_1_- *vs.* β_2_-selectivity (9–11). Unfortunately, this results in β_2_-AR blockade in the airways that risks, in susceptible individuals, β_2_-mediated bronchospasm and reduced effectiveness of life-saving β_2_-agonist bronchodilation therapy (12). A meta-analysis of randomized controlled trials of β-blockers in those with asthma reports a significant fall in lung function [forced expiratory volume in 1 s (FEV1)], an increase in symptoms, and attenuation of β_2_-agonist rescue with cardioselective and nonselective β-blockers (13). Although the average fall in lung function was greater with nonselective agents, a >20% reduction in FEV1 still occurred in 1 in 8 patients who received cardioselective β-blockers. Other large, recent observational studies also report that both cardioselective and noncardioselective β-blockers result in reduced lung function in the general population [that recovers on cessation of β-blockers (14)] and in those with asthma when using β-blocker eye drops (15). A further observational study found that nonselective, but not cardioselective, β-blockers increase the risk of moderate and severe asthma exacerbations (16). In addition, catastrophic outcomes have been also reported (17–23). Taken together, these studies suggest that β_1_-selective (cardioselective) β-blockers are better than nonselective β-blockers, but that current cardioselective agents still pose a risk to those with asthma. β-Blockers are therefore contraindicated in patients with asthma (24).

Patients with chronic obstructive pulmonary disease (COPD) have a high risk of heart disease: 40% of those who suffer from COPD also have heart disease (25) and they have high cardiovascular mortality (26, 27). As up to 50% of patients with COPD also have significant airway reversibility (improvement in lung function with β-agonists), they are at risk of bronchospasm from β-blockers (28, 29). β-Blockers reduce lung function in COPD, often by more than the 5 to 10% that is suggested by the American Thoracic Society/European Respiratory Society as important in COPD, but this is often well tolerated [for full details, see Baker and Wilcox (7)]. There are no randomized placebo-controlled studies of β-blockers in patients with heart disease and COPD, but currently, the number of observational studies with favorable or neutral outcomes outweigh those with detrimental outcomes (7, 30–32). Because of high cardiovascular mortality, current guidelines recommend trying β-blockers under careful medical supervision for those with heart disease and COPD, although this is not without patient risk and causes significant clinician anxiety (33, 34).

Highly β_1_-selective antagonists would overcome the problems that are associated with unwanted β_2_-blockade. CGP20712A and LK204-545 are the two most highly β_1_-selective β-blockers reported to date, but neither was developed into clinical drugs (35, 36).

Partial agonism [intrinsic sympathomimetic activity (ISA)] also varies significantly between β-blockers. This was once considered beneficial: Partial agonists would block HR surges from adrenaline but also provide increased basal tone in heart failure and reduce the chance of bronchospasm (12); however, partial agonism has been proven to be detrimental. In heart failure, β-blockers with little or no ISA prolonged life (3, 4), whereas those with higher ISA had no benefit or were detrimental (37, 38). Post–myocardial infarction, β-blockers without ISA reduced mortality (5, 6), whereas those with ISA did not (39, 40). Lowering HR is required for increased survival (41). LK204-545 has significant β_1_-partial agonism (42).

Thus, highly β_1_-AR–selective antagonists, devoid of partial agonism, could overcome unwanted β_2_-blockade, increasing the safety of β-blockers in those with COPD and peripheral vascular disease and potentially allowing treatment in those with asthma. This study aimed to develop highly β_1_-selective orally bioavailable β-blockers with no agonism or off-target effects that also had pharmacokinetic (PK) properties suitable for once daily oral dosing.

## MATERIALS AND METHODS

### Study approval

Cardiovascular monitoring procedures were carried out with approval of the University of Nottingham Ethical Review Committee under Home Office Project and Personal License Authority (to S.M.G. and J.W.).

### NDD compounds

NDD-713 {4-[2-[[(2*S*)-3-[[(2*R*)-2-[[(cyclopropylmethoxy)methyl]-2,3-dihydro-1,4-benzodioxin-6-yl]oxy]-2-hydroxypropyl]amino]ethoxy]benzamide; Chemical Abstracts Survey (CAS) registry no.1392488-35-5} and NDD-825 {5-[2-[[(2*S*)-3-[[(2*R*)-2-[[(cyclopropylmethoxy)methyl]-2,3-dihydro-1,4-benzodioxin-6-yl]oxy]-2-hydroxypropyl]amino]ethoxy]-2,3-dihydro-1*H*-isoindol-1-one; CAS registry 1392488-42-4} were prepared and characterized as previously described (43).

### Cell culture

Chinese hamster ovary (CHO) cells that stably express human β_1_-ARs (CHO-β1: 1146 fmol/mg protein), human β_2_-ARs (CHO-β2: 466 fmol/mg protein), or human β_3_-ARs (CHO-β3: 790 fmol/mg protein) were grown as previously described (11). Human bronchial smooth muscle cells were obtained from Lonza (Basel, Switzerland) and grown in manufacturer media. Rat β-AR binding experiments (DNA from cDNA Resource Centre, Bloomsburg, PA, USA) were conducted by using transiently transfected CHO cells (44).

### ^3^H-CGP12177 whole-cell binding

Confluent cells were examined (96-well plates, 2 h incubation at 37°C) as previously described (11) with ^3^H-CGP12177 at 0.43–3.03 nM. Propranolol (10 μM) defined nonspecific binding. *K*_d_ values were derived from IC_50_ values by using the Cheng-Prusoff equation as previously described (11). *K*_d_ values for ^3^H-CGP12177, which were obtained from saturation binding in this study, were 0.49 ± 0.02 nM (*n* = 14) for human β_1_-AR; 0.28 ± 0.01 nM (*n* = 14) for human β_2_-AR; 0.24 ± 0.03 nM (*n* = 7) for rat β_1_-AR, and 0.25 ± 0.04 nM (*n* = 7) for rat β_2_-AR.

### ^3^H-cAMP accumulation

Confluent cells were examined (24-well plates, 5 h incubation at 37°C) as described in Baker (45). Antagonist *K*_d_ values were calculated from a rightward shift of the agonist concentration response curve by using the Gaddum equation as previously described (46).

### cAMP response element–secreted placental alkaline phosphatase reporter gene assay

cAMP response element (CRE)–secreted placental alkaline phosphatase (SPAP) production was measured in confluent cells (96-well plates, 5 h incubation at 37°C) as described in Baker (46). This assay is a sensitive measure of both cAMP and ERK1/2 MAPK pathways (47).

### Direct measurement of MAPK stimulation

MAPK stimulation was examined by using the AlphaScreen Surefire ERK1/2 T202/Y204 assay kit from TGR Biosciences (PerkinElmer, Waltham, MA, USA), which gives a direct quantitative readout of ERK1/2 (p42/44) MAPK phosphorylation. Confluent cells (half-well 96-well plates) were serum starved for 24 h, then the assay was performed per manufacturer instructions (5- and 10-min incubations at 37°C: ligand concentrations of 3 and 10 μM). Phorbol 12,13-dibutyrate (1 μM) was used as a positive control.

### Duration of ligand binding

A method that involved ligand washout was used as described (44). Cells were incubated with either competing ligand and ^3^H-CGP12177 for 2 h (control plate), or competing ligand alone for 2 h (duration plate). After 2 h, the control plate was processed while the duration plate was washed, and ^3^H-CGP12177 alone was added to wells and incubated for a second 2 h before processing. Long-acting ligands that do not dissociate from the receptor should result in similar ^3^H-CGP12177 binding to control. Shorter-acting ligands that were removed and/or that continued to dissociate during the 2-h ^3^H-CGP12177 incubation would result in more ^3^H-CGP12177 binding than control and, thus, an apparent rightward shift of the concentration response curve. Relative measures of ligand duration of binding are indicated by the degree of rightward shift of displacement curves (44).

### *In vitro* receptor selectivity profiling

Receptor selectivity of reference compounds LK204-545 and CGP20712A (10 μM), and NDD-713 (4.8 μM) and NDD-825 (2.4 μM; concentrations approximately 400-fold greater than β_1_ *K*_d_), were examined. *K*_i_ (inhibition constant) was calculated by using the Cheng-Prusoff equation from the mean of duplicate determinations in cell-based receptor radioligand-binding assays. Cerep *in vitro* pharmacology profiling service was used (see the Supplemental Data for a list of the assays used).

### Aqueous solubility

NDD-713.HCl and -825.HCl were added to either aqueous phosphate buffer (pH 6.5) or 0.01 M aqueous HCl (pH ∼2) at final DMSO concentrations of 1%. Samples were analyzed by nephelometry to determine solubility ranges (48). Studies were carried out by the Centre for Drug Candidate Optimisation (CDCO; Monash University, Melbourne, VIC, Australia).

### Lipophilicity

Octanol–water partition coefficient values (logD) of NDD-713 and -825 were estimated as described in Lombardo *et al.* (49) by correlation of the chromatographic retention properties of compounds against the characteristics of standard compounds with known partition coefficient values. Studies were carried out by CDCO.

### Permeability

CaCo-2 cells were grown in Transwell permeable supports, and confluency was assessed by transepithelial electrical resistance measurements (>270 Ω/cm^2^) and by unidirectional permeability (apical-basolateral) of the low- and high-permeability reference compounds, ^14^C-mannitol and ^3^H-propranolol. NDD-713.HCl and -825.HCl (20 µM in HBSS that contained 20 mM HEPES, pH 7.4) were transferred to the donor compartment of Transwell plates and the acceptor compartments were filled with blank HBSS. Permeability was determined over 90 min by sampling both compartments. Quantitation of compound concentrations in donor and acceptor samples was by liquid chromatography–mass spectrometry (LC-MS; lower limit of quantification, 0.5–1.5 ng/ml), and for ^14^C-mannitol and ^3^H-propranolol, by liquid scintillation counting. Apparent permeability coefficients (*P*_app_) were calculated as follows: *P*_app_ (cm/s) = *dQ*/*dT* × 1/(*C*_0_ × *A*), where *dQ*/*dt* = apparent steady-state transport rate (μmol/s); *C*_0_ = initial concentration in the chamber (μmol/cm^3^); and *A* = surface area of CaCo-2 monolayer (0.3cm^2^). Efflux ratios were calculated as the ratio of mean *P*_app_ values in B→A and A→B directions. Studies were carried out by CDCO.

### Metabolic stability

NDD-713.HCl and -825.HCl were incubated (0.02, 0.1, or 0.5 μM at 37°C, 120 min) with human (XenoTech, Kansas City, KS, USA) or rat liver microsomes (BD Biosciences, Brea, CA, USA) at 0.4 mg/ml protein concentration. Metabolic reaction was initiated by addition of NADPH-regenerating system or NADPH-regenerating system with uridine diphosphate glucuronic acid. Samples were quenched at time points by acetonitrile addition. Control samples, which contained neither NADPH, nor uridine diphosphate glucuronic acid, were monitored for degradation. Compound concentrations were determined by ultraperformance LC-MS (UPLC-MS) relative to calibration standards that were prepared in quenched liver microsomes and fitted to exponential decay functions to determine first-order rate constants (*k*) for substrate depletion. These were used to calculate *in vitro* intrinsic clearance (CL_int_) values: CL_int_ = *k*/microsomal protein content (0.4 mg protein/ml). Metabolic stability assays that used rat liver cytosol (BD Biosciences), human cryopreserved hepatocytes, or rat hepatocytes were carried out similarly. Average viable cell concentrations were determined by Trypan Blue exclusion methods (in the absence of test compounds). Studies were carried out by CDCO.

### Metabolism

NDD-713.HCl and -825.HCl (2 μM) and cytochrome P450 (P450) reference substrates (see Supplemental Data for full details) were incubated (37°C) with selected recombinant human P450 isoforms (CYP1A2, CYP2A6, CYP2B6, CYP2C8, CYP2C9, CYP2C19, CYP2D6 CYP2E1, and CYP3A4) in heterologously expressed P450 Supersomes (BD Biosciences) that were suspended in 50 M Tris-HCl buffer (pH 7.4; for CYP2C9) or 100 mM phosphate buffer (pH 7.4; for the other isoforms) at final P450 concentrations of 85 pmol/ml. Reactions were initiated by additions of an NADPH-regenerating system and quenched at time points over 60 min (reference probes) or 120 min (test compounds) by acetonitrile. Quenched samples were centrifuged and supernatants were collected. NDD-713, -825, and probe metabolites were determined by UPLC-MS relative to calibration standards prepared in quenched matrix, and %substrate that remained was calculated relative to the initial sample concentration. The %substrate that remained *vs*. time was fitted to exponential decay functions to determine the pseudo first-order rate constant (*k*/min) for substrate depletion. These were normalized to P450 concentrations (pmol/ml) to obtain *in vitro* CL_int_ values: CL_int_, *in vitro* (μl/min/pmol P450) = [*k* (min^−1^)/P450 concentration (pmol/min)] × 1000. Studies were carried out by CDCO.

### P450 inhibition

Concentrations of NDD-713.HCl and -825.HCl (0.25–20 μM) were incubated in triplicate (37°C for 4–40 min) with substrates specific for individual P450 isoforms (see Supplemental Data for full details) in human liver microsomes (batch 34689 for CYP1A2 and CYP3A4; batch 0910251 for CYP2C9, CYP2C19, and CYP2D6) at protein concentrations of 0.4 mg/ml (CYP1A2, CYP2C9, CYP2D6 and CYP3A4/5) or 1.0 mg/ml (CYP2C19) and a total organic solvent concentration <1%. Reaction was initiated by addition of NADPH-regenerating system and samples were quenched by addition of ice-cold acetonitrile, which contained 0.15 μg/ml diazepam as an internal standard, before determination of the concentration of specific metabolites by LC-MS. Compound IC_50_ values (and positive control) for each P450 isoform was assessed according to %reduction in formation of the specific metabolite (maximal metabolite formation occurring in the absence of inhibitor). IC_50_ was taken as the concentration that caused a 50% reduction of the metabolite formed relative to the maximal extent of formation. Control samples were included to confirm that the LC-MS assay was not affected by test compounds (and potential metabolites). Studies were carried out by CDCO.

### Protein binding

Protein binding of 1 µM NDD-713.HCl and -825.HCl in human and rat plasma, as well as human liver microsomes, was assessed by using the ultracentrifugation technique. Frozen human plasma (Australian Red Cross Blood Service, Melbourne, VIC, Australia) and rat plasma (male Sprague-Dawley rats; Charles River, Margate, Kent, United Kingdom) were thawed at 37°C. Human liver microsomes (XenoTech) were suspended in 0.1 M phosphate buffer (pH 7.4) at protein concentrations of 0.4 mg/ml. Samples were collected immediately as stability reference controls. Spiked plasma samples (but not microsome samples) were incubated at 37°C for 1 h. Aliquots of the spiked matrix were spun (223,000 *g*, Beckman rotor type 42.2Ti; Beckman Coulter, Brea, CA, USA) for 4.2 h at 37°C to separate proteins. Noncentrifuged control samples were maintained at 37°C. After ultracentrifugation, supernatant was stored frozen, together with noncentrifuged samples at −20°C. NDD-713.HCl and -825.HCl concentrations of noncentrifuged matrix and protein-free supernatant were determined by UPLC-MS relative to calibration standards prepared in the respective matrices. Total concentration (*C*_total_) was the average measured concentration in centrifuged samples maintained at 37°C, and unbound concentration (*C*_unbound_) was the average measured concentration in protein-free supernatants from centrifuged samples (*n* = 3). The compound unbound fraction (*f*_u_) was calculated: *f*_u_ = *C*_unbound_/*C*_total_. Compound degradation was assessed by comparing concentrations in noncentrifuged plasma samples that were incubated at 37°C with concentrations measured in the *t* = 0 noncentrifuged samples. Plasma protein binding values were estimated by correlation of their chromatographic retention properties on human albumin columns against those of standard compounds with known protein binding values as previously described (50). Studies were carried out by CDCO.

### PK analysis

NDD-713.HCl and -825.HCl were intravenously administered after overnight food withdrawal to male Sprague-Dawley rats (weighing 241–289 g) at 2 or 6 mg/kg (formulated at 1 mg/ml in aqueous vehicle that contained 5% DMSO and 5% glucose) or 20 mg/kg (formulated at 5.3 mg/ml in aqueous vehicle that contained 5% DMSO in 0.2 M Captisol; Ligand Pharmaceuticals, Inc., San Diego, CA, USA) as a 10-min constant-rate infusion (1 ml/rat, 3 rats/dose level) and orally at 2, 10, or 20 mg/kg (formulated at 0.4–2.3 mg/ml in aqueous vehicle that contained 0.5% hydroxymethylcellulose, 0.5% benzyl alcohol, and 0.4% Tween-80) by gavage (1.0 ml/rat, 3 rats/compound). Arterial blood was collected for 24 h directly into borosilicate vials (at 4°C) that contained heparin, Complete (a protease inhibitor cocktail; Sigma-Aldrich, St. Louis, MO, USA), potassium fluoride, and EDTA to minimize potential *ex vivo* degradation of compounds in blood and plasma samples. Blood samples were centrifuged, supernatant plasma was removed, and plasma concentrations of compounds were determined by LC-MS. Bioavailability was calculated relative to the mean AUC value for each compound observed after intravenous dosing at 2 mg/kg. Studies were carried out by CDCO.

### *In vivo* pharmacology

Adult male Sprague-Dawley rats, weighing 300−400 g, were chronically instrumented with pulsed Doppler flow probes and intravascular catheters for cardiovascular monitoring and drug administration in a 2-stage surgical protocol as described (51). Surgery was performed under general anesthesia (fentanyl and medetomidine, 300 μg/kg of each, i.p.), with reversal and postoperative analgesia provided by atipamezole (1 mg/kg, s.c.) and buprenorphine (0.02 mg/kg, s.c.). Experiments were performed with animals that were fully conscious and unrestrained in home cages and given free access to food and water. Cardiovascular variables were recorded by using a customized, computer-based system (Instrument Development Engineering Evaluation, Maastrich Instruments Bv, Maastrich, The Netherlands) with raw data sampled every 2 ms. Hindquarters vascular conductance (HQC) was calculated as Doppler shift (flow)/mean arterial pressure. Throughout, atropine methyl nitrate (1 mg/kg/h) was infused (0.4 ml/h) continuously to inhibit vagally induced HR changes.

#### Intravenous administration of β-blocker

Starting at least 1 h after atropine infusion began, rats were intravenously administered 3 doses of isoprenaline [12, 40, and 120 ng/kg/min, 3-min infusions (0.15 ml/min), 20–30 min between doses], before a β-blocker was administered as an intravenous bolus (2 mg/kg, 0.1 ml, except CGP20712A, which was 200 μg/kg; in saline) that was maintained by continuous infusion for 90 min (1 mg/kg/h, 0.4 ml/h, except CGP20712A, which was 100 μg/kg/h). Isoprenaline infusions were repeated during the final 60 min of β-blocker infusion and 3–4 and 23–24 h after β-blocker infusions finished.

#### Oral administration of increasing doses of NDD-825.HCl

Experiments ran over 3 consecutive days. Lightly sedated rats (propofol at 0.1–0.2 ml of 10 mg/ml, i.v.) that were instrumented for cardiovascular monitoring were administered an oral dose of NDD-825.HCl (1 mg/kg on d 1, 3 mg/kg on d 2, 10 mg/kg on d 3; 10 ml/kg in water) by oral gavage. Each day, responses to isoprenaline infusions were monitored before and at 0.5–1.5 and 3–4 h after oral NDD-825.HCl administration.

Isoprenaline responses shown are absolute values for HR and HQC before the first dose of isoprenaline (baseline) and during the third minute of infusion of each dose. Values at baseline and during the highest isoprenaline dose were tested for statistical significance by using a nonparametric ANOVA test with correction for repeat measures [Quade test (52)]. A value of *P* < 0.05 was taken as significant.

### Cytotoxicity

Human Caucasian hepatocyte carcinoma cells (HepG2; European Collection of Cell Cultures, Salisbury, United Kingdom) were grown in accordance with their guidelines by using DMEM. Cells at a density of 30,000 cells/well in 96-well plates were incubated overnight (37°C, 5% CO_2_ atmosphere). Medium was then removed and replaced with DMEM that contained control or compounds (1, 10, and 100 μM; *n* = 2 per concentration) for 24 h before addition of 100 μl/well of CellTiter-Glo Reagent (Promega, Madison, WI, USA) for ATP measurement and shaken to induce cell lysis. Luminescence was quantified on an AnalystAD plate-reader (Molecular Devices, Sunnyvale, CA, USA). Thioridizine (known HepG2 cytotoxicity) was used as positive control. Study was carried out by BioFocus DPI (Saffron Walden, United Kingdom).

### hERG inhibition

Binding to the hERG potassium channel (recombinant protein from HEK293 cells) was examined by using ^3^H-astemizole radioligand binding assays [Cerep (53)] by measuring the displacement of radioligand binding from the channel by 10 µM NDD-713 and -825. In addition, the potential of the compound to inhibit hERG channels was tested by using an automated patch-clamp electrophysiological assay (BioFocus, Cambridge, United Kingdom; using the Precision hERG-HEK recombinant cell line from on an Ion Works Quattro automated electrophysiology platform; Millipore, Billerica, MA, USA). The antiarrhythmic research drug E4031 was used as positive control (IC_50_ = 83 nM).

### Ames genotoxicity

Ames fluctuation assays were performed by using *Salmonella typhimurium* strains TA98, TA100, TA1535, and TA1537 to detect frame-shifts and base substitutions that led to missense mutations in liquid culture in 384-well plates. Bacterial plates were incubated with serially diluted NDD-713 and -825 solutions for 96 h, and bacterial growth was measured spectrophotometrically by using a pH color-change indicator in response to bacterial growth acidification. Assays were conducted with or without metabolic activation *via* addition of rat liver S9 fraction. Bacteriocidal or bacteriostatic effects were assessed by using a bacterial cytotoxicity assay that was conducted in parallel with Ames fluctuation assays. Reference compounds were 2-aminoanthracene, 9-aminoacridine, quercetin, streptozotocin (genotoxicity), and mitomycin C (bacterial cytotoxicity). Study was carried out by Cerep (Celle L’Evescault, France).

### Seven-day oral dose range rat toxicity study

Male Crl:CD rats (9 wk old, weighing 316.8–349.4 g on d 1; Charles River) were administered 1 oral dose of NDD-825.HCl in water that contained 0.5% (w/v) hydroxypropylmethylcellulose K15M, 0.5% (v/v) benzyl alcohol, and 0.4% (v/v) Tween-80 at 20, 90, or 300 mg/kg at a dose volume of 10 ml/kg for 7 consecutive days (4 animals per group). Blood samples were collected before and at time points during the 24 h that followed for bioanalysis. Animals were monitored for adverse clinical signs and body weight. Terminal blood samples were taken for clinical chemistry, and organs were harvested for microscopic examination (see Supplemental Data for full details). Three additional male rats per group received NDD-825.HCl on d 1 only to collect blood samples for plasma bioanalysis of compound toxicokinetics. NDD-825 was quantifiable in plasma of all animals up to 8 h after dose administration; *t*_max_ occurred between 2 and 8 h after dosing. Systemic exposure to NDD-825 in terms of *C*_max_ increased in approximate proportion with increasing dose, whereas for the same dose increase, AUC_0-*t*_ increased in a supraproportional manner across the entire dose range. Overall, for a 15-fold increase in dose from 20 to 300 mg/kg/d, AUC_0-*t*_ and *C*_max_ increased 31.4- and 12.4-fold, respectively. After repeat daily oral administration of NDD-825 for 7 d, systemic exposure in terms of both *C*_max_ and AUC_0-*t*_ was comparable to that on d 1 at each respective dose level. Study was carried out by Aptuit (Verona, Italy).

### Calculation of selectivity indices

Selectivity indices (SIs) for bisoprolol in terms of receptor binding (SI_1_; Supplemental Eq. 1) and receptor occupancy (SI_2_; Supplemental Eq. 2) were determined on the basis of the following parameters: *K*_d_ values at the human βARs from ^3^H-CGP12177 whole-cell binding (**Table 1**) for SI_1a_; *K*_d,β_ values from antagonism of cimaterol-induced ^3^H-cAMP accumulation (**Table 2**) for SI_1b_; and *C*_ssf_ (at 10 mg/d p.o.) = 87.3 nM (23). SI_2_ values for NDD-713 and -825 were estimated similarly (using SI_1a_ data) and assuming that as the bisoprolol dose (10 mg/d, p.o.) required to lower exercise-induced HR is 35-fold over *K*_d_ (bisoprolol *C*_ssf_/*K*_d,β1AR_ = 35) (23), a similar 35-fold-higher dose of NDD-713 and -825 would also be required. *In vivo* pharmacologic responses in humans (*E*_β-AR_) were calculated for both β-ARs by using Supplemental Eq. 3, where *E*_β1-AR_ and *E*_β2-AR_ refer to effects on exercise HR and on FEV1 under steady-state conditions after drug administration, respectively. Supplemental Eq. 3 is based (23, 54) on a ternary complex model (Supplemental Eqs. 4–6) of agonist [*A*], receptor [*R*], transducer element [*T*], antagonist [*B*], total receptor [*R*_0_], and transducer [*T*_0_] concentrations are based on Supplemental Eqs. 7 and 8. The following values (23, 54) for β_1_-AR were used for the analysis: *E*_β-AR,max_ = 35%, *K*_DA_ = 2 μM, and [*A*] = 0.02 μM. For β_2_-AR, we used *E*_β-AR,max_ = 50%, *K*_DA_ = 3 μM, and [*A*] = 0.01 μM. The values (23, 54) used for the analysis of exercise HR were *K*_DAR_ = 0.64 nM, [*R*_0_] = 0.29 nM, and [*T*_0_] = 700 nM. The corresponding values (23, 54) used for the analysis of FEV1 were *K*_DAR_ = 2.6 nM, [*R*_0_] = 1.1 nM, and [*T*_0_] = 2.6 μM, respectively. SI values in terms of *in vivo* human pharmacologic response were calculated by using Supplemental Eq. 9.

**TABLE 1.** Affinity and selectivity of existing clinically used and novel β-AR ligands

Compound	Human β_1_-AR		Human β_2_-AR		Human β_1_ *vs*. β_2_ selectivity
	Log *K*_d_	*n*	Log *K*_d_	*n*	*K*_d,β2_/*K*_d,β1_
Bisoprolol	−7.96 ± 0.03	16	−6.33 ± 0.03	15	42.7
Carvedilol	−9.19 ± 0.04	10	−9.83 ± 0.06	5	0.23
CGP20712A	−8.76 ± 0.03	94	−5.62 ± 0.02	66	1380
ICI118551	−6.66 ± 0.02	88	−9.17 ± 0.02	66	0.003
LK204-545	−8.17 ± 0.07	15	−5.30 ± 0.04*^a^*	11	741
NDD-713	−7.82 ± 0.03	27	−5.05 ± 0.03*^a^*	23	589
NDD-713.HCl	−7.70 ± 0.04	24	−5.01 ± 0.04*^a^*	18	490
NDD-825	−8.28 ± 0.05	20	−5.27 ± 0.03*^a^*	16	1023
NDD-825.HCl	−8.17 ± 0.03	21	−5.34 ± 0.05*^a^*	16	676

	Rat β_1_-AR		Rat β_2_-AR		Rat β_1_ *vs*. β_2_ selectivity
Compound	Log *K*_d_	*n*	Log *K*_d_	*n*	*K*_d,ratβ2_/*K*_d,ratβ1_
Bisoprolol	−8.24 ± 0.04	5	−5.82 ± 0.04	5	263
Carvedilol	−9.44 ± 0.07	6	−9.72 ± 0.06	6	0.52
CGP20712A	−9.12 ± 0.07	6	−5.15 ± 0.06	6	9322
ICI118551	−6.89 ± 0.04	6	−9.26 ± 0.06	6	0.003
LK204-545	−8.36 ± 0.05	6	−4.81 ± 0.04*^a^*	6	3548
NDD-713	−8.07 ± 0.09	5	−4.73 ± 0.08*^a^*	5	2188
NDD-825	−8.37 ± 0.02	6	−4.88 ± 0.02*^a^*	6	3090

Log *K_d_* values and ligand selectivities as determined from ^3^H-CGP12177 whole-cell binding in stable cells lines that express human receptors (CHO-β_1_ and CHO-β_2_ cells) or in CHO cells that are transiently transfected with the β_1_- or rat β_2_-AR. Values shown are means ± sem of *n* separate experiments. Thus, bisoprolol was 42.7 times more selective for human β_1_-AR than human β_2_-AR, whereas carvedilol was 4.4 times more β_2_-selective. *^a^*Apparent log *K*_d_ values: these are reported when the maximum concentration of competing ligand was not able to inhibit all of the specific binding (*e.g.*, NDD-825 and -713 in Fig. 2*B*). The apparent *K_d_* value given is therefore assuming that all specific binding would be inhibited by a higher concentration of competing ligand.

**TABLE 2.** Affinity and selectivity of existing clinically used and novel β-AR ligands as determined from antagonism of cimaterol-induced ^3^H-cAMP accumulation in CHO-β_1_, CHO-β_2_, or CHO-β_3_ cells

Compound	Human β_1_-AR				Human β_2_-AR		Human β_3_-AR		β_1_ *vs*. β_2_ selectivity
	Log *K*_d,β1_	*n*	Schild slope	*n*	Log *K*_d,β2_	*n*	Log *K*_d,β3_	*n*	*K*_d,β2_/*K*_d,β1_
Bisoprolol	−8.60 ± 0.03	15	0.97 ± 0.03	5	−6.76 ± 0.03	13	−5.65 ± 0.06	5	69
Carvedilol	−9.75 ± 0.12	12	1.29 ± 0.09	4	−10.62 ± 0.08	4	−8.54 ± 0.18	3	0.13
Nebivolol	−9.28 ± 0.08	22	1.15 ± 0.03	5	−7.91 ± 0.09	11	−6.35 ± 0.06	9	23
CGP20712A	−9.64 ± 0.03	15	1.03 ± 0.03	5	−6.02 ± 0.03	8	−5.47 ± 0.04	5	4169
NDD-713	−8.52 ± 0.03	12	1.06 ± 0.03	4	−5.44 ± 0.03	4	−5.47 ± 0.08	3	1202
NDD-825	−8.99 ± 0.05	9	1.04 ± 0.02	3	−5.75 ± 0.06	4	−5.72 ± 0.08	7	1738

The Schild slope is that obtained in CHO-β_1_ cells from the rightward shift of the cimaterol dose-response curve by 3 different concentrations of antagonist in the same experiment (*e.g.*, Fig. 3) where a value of 1 represents competitive antagonism. The affinity of most ligands at β_2_-AR and β_3_-AR was too low to allow a Schild plot to be constructed. Values shown are means ± sem of *n* separate experiments.

## RESULTS

**Figure 1.**
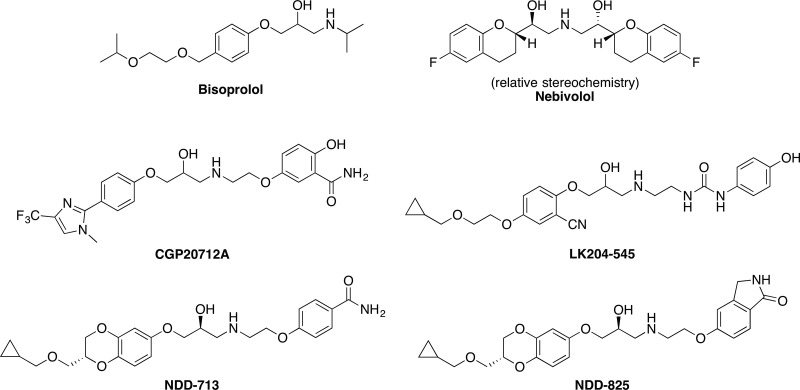
Chemical structures of β_1_-selective β-blockers.

As LK204-545 is a β_1_-selective ligand, we started with des-cyano analogs of LK204-545 (55), and after many iterations of multiparameter lead optimization, NDD-713 and -825 were the two compounds with the best overall performance (**Fig. 1**) (43).

### Molecular pharmacology of NDD-713 and -825

#### Affinity and duration of action

**Figure 2.**
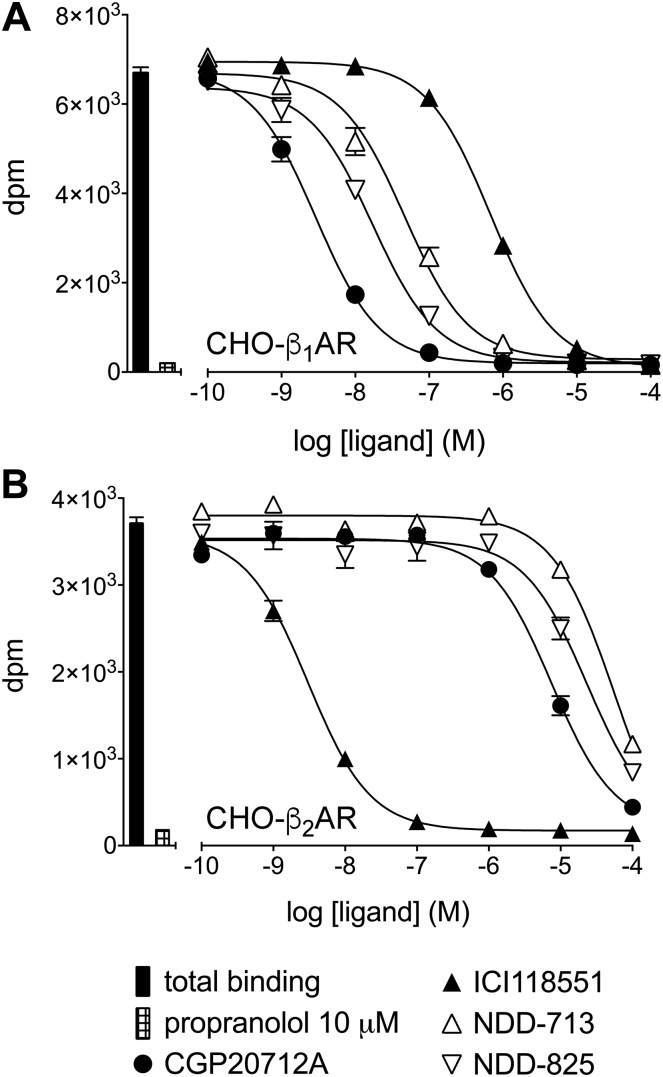
Inhibition of ^3^H-CGP12177 whole-cell binding in response to CGP20712A, ICI118551, NDD-713, and NDD-825 in CHO-β_1_ (*A*) and CHO-β_2_ (*B*) cells. Nonspecific binding was determined by 10 μM propranolol. Data points are means ± sem of triplicate determinations, and the concentrations of ^3^H-CGP12177 present in these experiments was 0.89 nM. These single experiments are representative of 20 (*A*) and 16 (*B*) separate experiments.

Ligand affinity for human β_1_- and β_2_-AR was assessed first by radioligand binding (**Fig. 2**). CGP20712A was a highly β_1_-selective ligand (1380-fold β_1_-selective) and ICI118551 a highly β_2_-selective ligand (333-fold β_2_-selective), whereas current clinically used β-blockers had relatively poor selectivity in comparison (Fig. 2 and Table 1). NDD-713 and -825 both had high β_1_-affinity but poor β_2_-affinity (Fig. 2 and Table 1). At human β_3_-AR, NDD-713 and -825 affinity was so low that accurate measurements were not possible. Affinities of compounds for rat β_1_- and β_2_-AR were also determined by using transiently transfected cells (Table 1).

**Figure 3.**
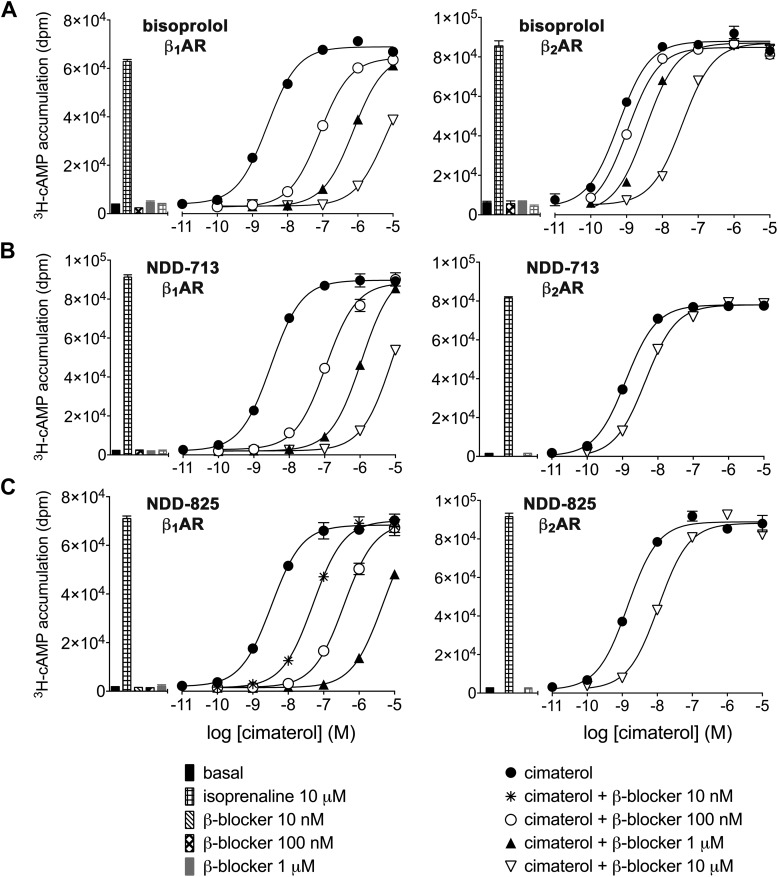
^3^H-cAMP accumulation in response to cimaterol in the absence or presence of bisoprolol (*A*), NDD-713 (*B*), and NDD-825 (*C*) in CHO-β_1_ and CHO-β_2_ cells. Bars represent basal ^3^H-cAMP accumulation and accumulation in response to 10 μM isoprenaline or β-blocker alone. Data points are means ± sem of triplicate determinations, and these single experiments are representative of 5 (*A*), 4 (*B*), and 3 (*C*) separate experiments in each case.

Ligand affinity was also assessed in a functional assay by examining the ability of ligands to inhibit cimaterol-induced ^3^H-cAMP accumulation responses (**Fig. 3** and Table 2). Cimaterol is a stable β-agonist—less susceptible to degradation over 5 h at 37°C than adrenaline or noradrenaline—that is active across all 3 β-AR subtypes and that activates high-affinity catecholamine conformation of the β_1_-AR (42, 55). NDD-713 and -825 readily inhibited β_1_-mediated cimaterol-induced responses but not β_2_- or β_3_-mediated responses, which confirmed high human β_1_-affinity and high β_1_-selectivity over β_2_- and β_3_-ARs.

Finally, the ligand affinity for human β_2_-AR was also determined in the more native environment of human bronchial smooth muscle cells. Here, cimaterol-induced ^3^H-cAMP was readily inhibited with ICI118551 but poorly inhibited by CGP20712A, NDD-713, and -825 (**Table 3**). Thus, NDD-713 and -825 had poor affinity for native β_2_-ARs in these airway cells.

**TABLE 3.** Affinity values obtained for ligands in human bronchial smooth muscle cells (that natively express β_2_-AR) and in CHO-β_2_ cells that stably express a transfected human β_2_-AR determined from antagonism of cimaterol-induced ^3^H-cAMP accumulation

Antagonist	Human bronchial smooth muscle cells		CHO-β_2_ cells	
	Log *K*_d_	*n*	Log *K*_d_	*n*
CGP20712A	−6.06 ± 0.08	4	−6.02 ± 0.03	8
ICI118551	−9.51 ± 0.13	4	−9.65 ± 0.03	6
NDD-713	−5.47 ± 0.05	4	−5.44 ± 0.03	4
NDD-825	−5.87 ± 0.11	4	−5.75 ± 0.06	4

Log *K_d_* values shown are means ± sem of *n* separate experiments.

To assess whether β_1_-AR–antagonist interactions were competitive, Schild plots (dose ratio −1 *vs.* ligand concentration) (56) were constructed from experiments with 3 different concentrations of antagonists (Fig. 3). A slope value of 1 represents competitive antagonism and this was observed with all antagonists (Table 2). The affinity of NDD-713 and -825 was too low for this to be determined at β_2_- and β_3_-ARs.

**Figure 4.**
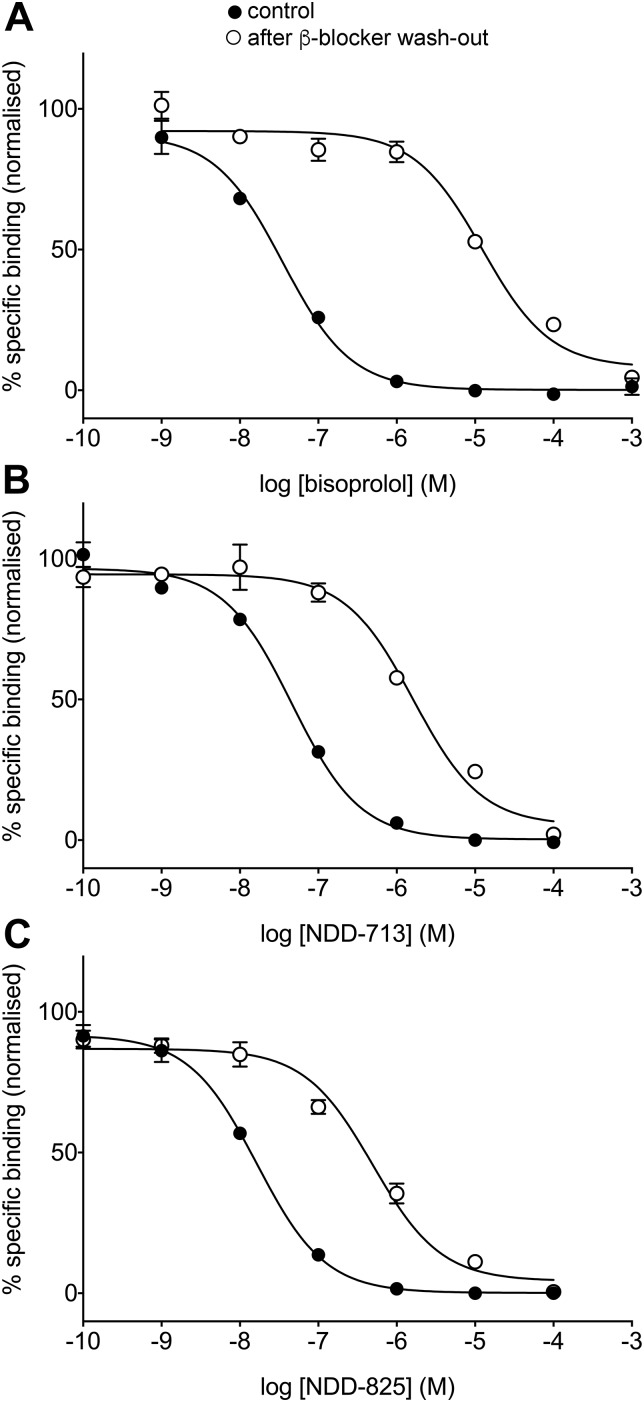
Inhibition of specific ^3^H-CGP12177 whole-cell binding in CHO-β_1_ cells in response to bisoprolol (*A*), NDD-713 (*B*), and -825 (*C*), with the control curve (solid circles) and after washout of the competing β-blocker (open circles) as described in Materials and Methods. Nonspecific binding was determined by 10 μM propranolol. Data points are means ± sem of triplicate determinations, and the concentrations of ^3^H-CGP12177 present in these experiments were 0.81 nM (*A*), 0.96 nM (*B*), and 0.74 nM (*C*). These single experiments are representative of 4 (*A*), 9 (*B*), and 8 (*C*) separate experiments.

An indication of duration of receptor occupancy was sought by using a washout technique (44) where ligand residence time is indicated by a rightward shift of the washout curve relative to control (**Fig. 4** and **Table 4**). Carvedilol had a long residence time (control and washout curves are similar; log shift of 0.3 = 2-fold), which indicated no β_1_-AR dissociation. In contrast, bisoprolol was readily washed out (rightward log shift of 2.7; 501-fold), which indicated significant ligand dissociation. NDD-713 and -825 had intermediate residence times (Fig. 4 and Table 4).

**TABLE 4.** Relative duration of binding of existing clinically used and novel β-AR ligands as determined from ^3^H-CGP12177 whole-cell binding in CHO-β_1_ cells

Compound	Human β_1_-AR	
	Log shift	*n*
Bisoprolol	2.70 ± 0.18	4
Carvedilol	0.31 ± 0.06	5
CGP20712A	1.35 ± 0.04	28
ICI118551	1.64 ± 0.07	24
Nebivolol	0.26 ± 0.07	3
LK204-545	2.06 ± 0.08	6
NDD-713	1.75 ± 0.08	9
NDD-825	1.62 ± 0.11	8

The log shift given is the measure of the rightward shift of the washout curve compared with the control curve examined in parallel in each experiment as seen in Fig. 4. A greater shift represents shorter duration ligands, and smaller shifts represent longer duration ligands. Values shown are means ± sem of *n* separate experiments (*n*).

#### Intrinsic efficacy

**Figure 5.**
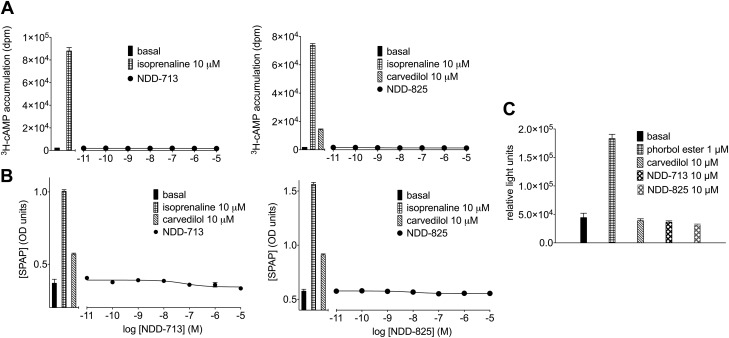
*A*) ^3^H-cAMP accumulation in CHO-β_1_ cells in response to NDD-713 and -825. Bars represent basal ^3^H-cAMP accumulation, in response to 10 μM isoprenaline alone, and in response to 10 μM carvedilol alone. Data points are means ± sem of triplicate determinations, and these single experiments are representative of 8 separate experiments in each case. *B*) CRE-SPAP production in CHO-β_1_ cells in response to NDD-713 and -825. Bars represent basal CRE-SPAP production, in response to 10 μM isoprenaline alone, and in response to 10 μM carvedilol alone. Data points are means ± sem of triplicate determinations and are representative of 5 separate experiments in each case. *C*) Basal and ERK1/2 MAPK activation in CHO-β_1_ cells in response to phorbol ester, carvedilol, NDD-713, and -825 (5 min incubation). Bars are means ± sem of triplicate determinations. This single experiment is representative of 4 separate experiments.

Ligand efficacy was initially assessed at the primary G_s_-cAMP pathway. Carvedilol and nebivolol were weak partial β_1_-AR agonists, stimulating maximum responses of 15.2 ± 1.0% (*n* = 20) and 5.0 ± 0.8% (*n* = 4), respectively, of that with isoprenaline (similar to previous reports) (51). LK204-545 stimulated a response that was 70.1 ± 2.5% (*n* = 3) that of isoprenaline. No ^3^H-cAMP responses were observed with bisoprolol, CGP20712A, NDD-713, or -825 (**Fig. 5*A***). In other signaling pathways, carvedilol and LK204-545 stimulated CRE-SPAP production (a sensitive readout of both cAMP and ERK1/2 MAPK) that was 31.2 ± 2.8 and 79.3 ± 2.7% (*n* = 5), respectively of the isoprenaline maximum, whereas no responses were observed with bisoprolol, CGP20712A, nebivolol, NDD-713, or -825 (Fig. 5*B*). Similarly, no agonist responses were observed when directly measuring ERK1/2 phosphorylation (Fig. 5*C*).

Previous studies that examined responses in the parent cell line CHO-CRE-SPAP cells (*i.e.,* cells without the transfected receptor) have demonstrated that there is no endogenous β-AR present in these cells. There were no ^3^H-cAMP accumulation or CRE-SPAP gene transcription responses to a wide range of different ligands (45, 47).

#### Other GPCR and ion channel effects

**Figure 6.**
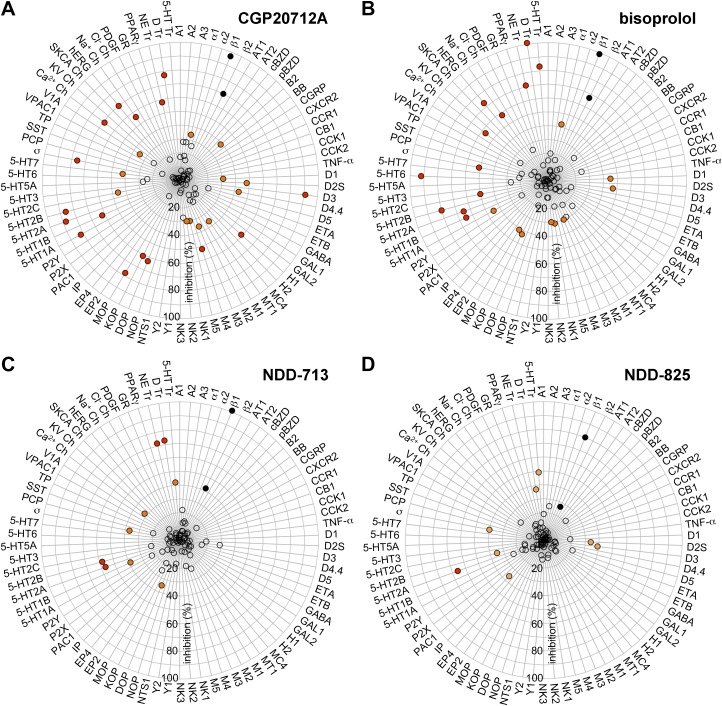
Receptor selectivity of CGP20712A (at 10 μM; *A*), LK204-545 (at 10 μM; *B*), NDD-713 (at 4.8 μM; *C*), and -825 (at 2.4 μM; *D*). The compounds were assayed against the receptors listed in duplicate and average values are shown. Values for β-ARs are shown as black circles, those for receptors with >50% inhibition as red circles, those in the range of 30–50% inhibition as orange circles, and those with <30% inhibition as open circles. GPCRs: adenosine (A1, A2, A3), adrenoceptor (α1, α2, β1, β2), angiotensin (AT1, AT2), benzodiazepine, central and peripheral (cBZD, pBZD), bombesin (BB), bradykinin (B2), chemokine (CGPR, CXCR2, CCR1), cannabinoid (CB1), cholecystokinin (CCK1, CCK2), cytokine (TNF-α), dopamine (D1, D1S, D3, D4.4, D5), endothelin (ETA, ETB), GABA, galanin (GAL1, GAL2), histamine (H1, H2), melanocortin (MC4), motilin (MT1), muscarinic acetylcholine (M1, M2, M3, M4, M5), neurokinin (NK1, NK2, NK3), neuropeptide Y (Y1, Y2), neurotensin (NTS1), nociception (NOP), opioid (DOP, KOP, MOP), prostaglandin (EP2, EP4, IP), pituitary adenylate cyclase-activating polypeptide (PAC1), purinergic (P2X, P2Y), serotonergic (5-HT1A, 5-HT1B, 5-HT2A, 5-HT2B, 5-HT2C, 5-HT3, 5-HT5A, 5-HT-6, 5-HT7), σ (σ, PCP), somatostatin (SST), thromboxane (TP), vasoactive intestinal polypeptide (VPAC1), vasopressin (V1A). Ion channels: calcium channel L verapamil site (Ca^2+^ Ch), potassium channel (KV Ch), sodium channels (SKCa Ch, hERG membrane preparation, Na^+^ Ch site 2), chloride channel (Cl^−^ Ch GABA-gated), Tyr kinase receptor (PDGF). Nuclear hormone receptors: glucocorticoid (GR), peroxisome proliferator (PPARγ). Monoamine transporter: noradrenaline (NE Tr), dopamine (D Tr), serotonin (5-HT Tr).

To assess potential off-target effects, CGP20712A, LK204-545, NDD-713, and -825 binding was assessed by using the Cerep *in vitro* pharmacology profiling service in a diverse panel of 80 receptors and ion channels (**Fig. 6**). Considerably more binding to other targets (off-target effects) was observed with CGP20712A and LK204-545 compared with NDD-713 or -825. Where NDD-713 or -825 receptor binding inhibition exceeded 50%, concentration–response experiments were conducted. *K*_i_ values obtained were as follows: NDD-713: *K*_i_ (5-HT2A), 7.7 μM; *K*_i_ (5-HT2B), >10 μM; *K*_i_ (noradrenaline transporter), 2.8 μM; *K*_i_ (dopamine transporter), 0.8 μM; and NDD-825: *K*_i_ (5-HT2A), 5.8 μM.

#### *In vitro* absorption, distribution, metabolism and elimination of NDD-713 and -825

*In vitro* disposition properties of NDD-713 and -825 were assessed in terms of solubility, lipophilicity, protein binding, cell permeability, metabolic stability, and P450 metabolism (**Table 5**). Both compounds are basic, display good pH-dependent aqueous solubility, and have comparatively low lipophilicity, plasma binding, and intrinsic clearance in both rat and human liver microsome and hepatocyte preparations. NDD-713 showed high and NDD-825 intermediate apical-to-basolateral membrane permeability in the bidirectional CaCo-2 assay, without pronounced efflux of either compound. NDD-713 was metabolized predominantly by CYP3A4 and CYP2A6 isoforms, whereas NDD-825 was metabolized mainly by CYP3A4 and CYP2D6 isoforms. Neither compound inhibited the activity of a panel of P450 enzymes.

**TABLE 5.** *In vitro* disposition properties of NDD-713 and -825

Parameter	Assay	Units	NDD-713	NDD-825
Lipophilicity	Distribution coefficient, pH 3	gLogD_pH 3_	1.7	1.5
	Distribution coefficient, pH 7.4	gLogD_pH 7.4_	2.3	2.0
Solubility*^a^*	Aqueous, at pH 2.0	*S*_aq_ (μg/ml)	>100	>100
	Aqueous, at pH 6.5	*S*_aq_ (μg/ml)	25–50	50–100
Permeability*^a^*	Bidirectional CaCo-2	*P*_app, A-B_ (10^−6^ cm/s)	25 ± 4	6.5 ± 0.5
	Mass balance	A-B (%)	76 ± 10	79 ± 11
	Efflux ratio	*P*_app, B-A_/*P*_app, A-B_	<3	<3
Protein binding	Plasma protein	PPB (%)	63.5	73.5
	Human plasma	*f*_u_	0.15	0.01
	Rat plasma	*f*_u_	0.16	0.11
	Human liver microsomes	*f*_u_	0.67	0.63
Metabolic stability	Human liver microsomes*^b^*	CL_int_ (μl/min/mg)	<0.16	<0.16
	Rat liver microsomes*^b^*	CL_int_ (μl/min/mg)	<0.1	0.18 ± 0.2
	Human hepatocytes*^b^*	CL_int_ (μl/min/10^6^ cells)	<6	6
	Rat hepatocytes*^b^*	CL_int_ (μl/min/10^6^ cells)	minimal	5
	Rat liver cytosolic fraction*^b^*	CL_int_ (μl/min/mg)	<1.4	<1.4–1.7
Metabolism*^a^*	P450 *in vitro* clearance*^c^*	CL_int_ (μl/min/pmol P450)	0.02,^*d*,*g*^ 0.07^*e*,*g*^	0.02,^*f*,*g*^ 0.17^*e*,*g*^
	P450 inhibition*^h^*	IC_50_ (μM)	>20	>20

Values and ranges quoted are averages of 2 (or 3 where means ± sd are shown) separate determinations. *^a^*The hydrochloride salts of NDD-713 and -825 were used. *^b^*In the presence of NAD^+^, NADPH, or in the absence of cofactors. *^c^*Assayed: CYP1A2, CYP2A6, CYP2B6, CYP2C8, CYP2C9, CYP2C19, CYP2D6, CYP2E1, CYP3A4. *^d^*CYP2A6. *^e^*CYP3A4. *^f^*CYP2D6 and CYP2E1. *^g^*NDD-713 and -825 were not observed to be metabolized by other P450 isoforms assayed. *^h^*CYP1A2, CYP2C9, CYP2C19, CYP2D6, CYP3A4/5.

### *In vivo* pharmacology of NDD-713 and -825

#### PK analysis

**Figure 7.**
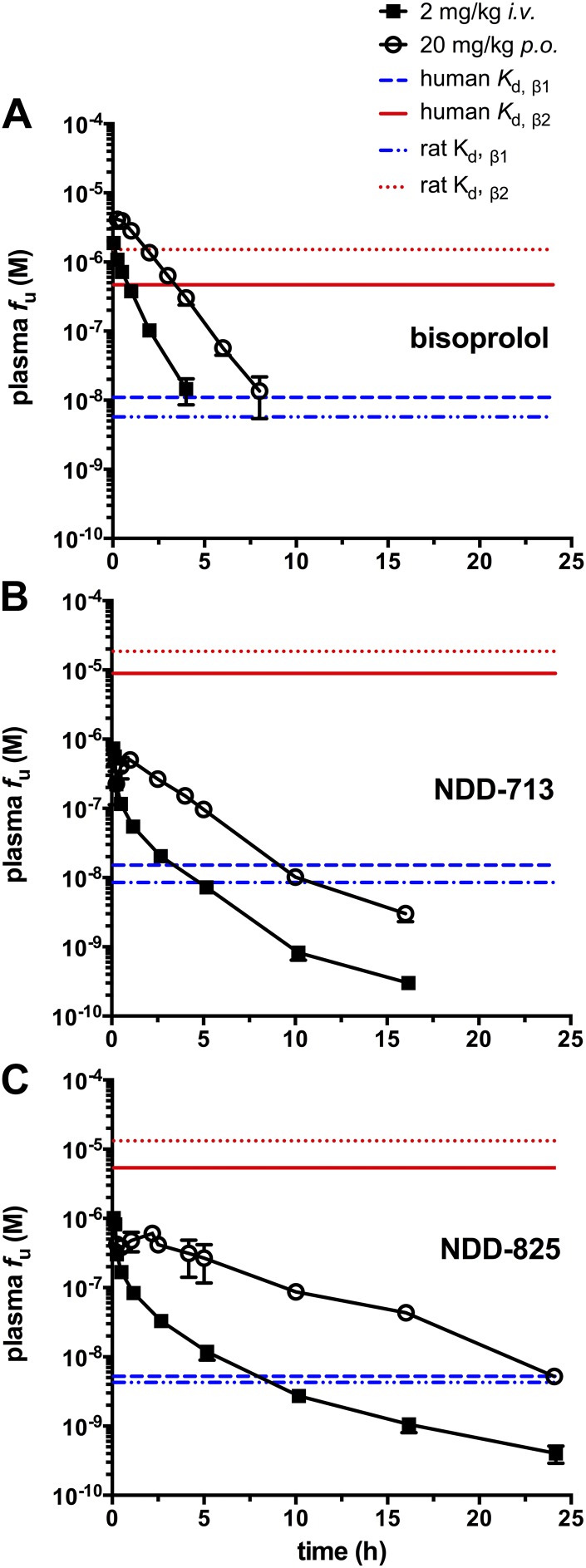
PK profiles of bisoprolol (*A*), NDD-713.HCl (*B*), and -825.HCl (*C*) after single bolus intravenous or oral. administration of compounds to rats. Plasma drug levels were corrected for plasma protein binding (see Table 5). Plasma *f*_u_ levels of compounds are plotted as a function of time postadministration (values are means ± sd; *n* = 3). Concentrations corresponding to human and rat β-AR *K_d_* values of the 3 compounds are shown as colored lines. Thus, to be effective, drug levels need to be above the blue lines to achieve β_1_-blockade, but below the red lines to avoid β_2_-blockade.

As the *in vitro* properties of NDD-713 and -825 (receptor affinity and disposition properties) were similar to those of bisoprolol (57), similar dosing levels were administered to rats. After a single dose of 20 mg/kg, p.o., the plasma *f*_u_ level of bisoprolol exceeded the β_2_-AR *K*_d_ value for >5 h, whereas those of NDD-713 and -825 never reached the β_2_-AR *K*_d_ (**Fig. 7**). PK analysis showed that bisoprolol was a short-acting agent, whereas NDD-825, in particular, revealed slow oral absorption and elimination alongside a large volume of distribution and, thus, was present at therapeutic concentrations for considerably longer (Fig. 7 and Tables 5 and **6**).

**TABLE 6.** PK properties of NDD-713 and -825 in rat after single bolus i.v. or p.o. administration (hydrochloride salt forms)

Parameter	Dose, i.v.					
	2 mg/kg		6 mg/kg		20 mg/kg	
	NDD-713	NDD-825	NDD-713	NDD-825	NDD-713	NDD-825
*t*_1/2, app_ (h)	1.7 ± 0.2	5.3 ± 0.3	1.6 ± 0.1	7.3 ± 0.1	2.0 ± 0	7.1 ± 1.3
Plasma CL_total_ (ml/min/kg)	62 ± 4	54 ± 2	43 ± 1	59 ± 1	51 ± 2	55 ± 1
Blood CL_total_ (ml/min/kg)	51 ± 3	39 ± 1	36 ± 1	42 ± 1	42 ± 1	39 ± 1
*V*_ss_ (L/kg)	4.3 ± 0.4	6.0 ± 0.5	3.6 ± 0.2	5.0 ± 0	4.2 ± 0.2	4.7 ± 0
AUC_0-*t*_ (μM ⋅ h)	1.2 ± 0.1	1.4 ± 0	4.6 ± 0.3	3.8 ± 0.2	14.7 ± 1.0	12.1 ± 0.3
AUC_0-*t*_/dose [μM ⋅ h/(mg/kg)]	0.6 ± 0.1	0.6 ± 0	0.8 ± 0	0.6 ± 0	0.7 ± 0	0.6 ± 0

	Dose, p.o.					
Parameter	2 mg/kg		10 mg/kg		20 mg/kg	
	NDD-713	NDD-825	NDD-713	NDD-825	NDD-713	NDD-825
*t*_1/2, app_ (h)	n.d.	9.8 ± 0.2	1.5 ± 0	4.6 ± 0.1	2.1 ± 0	3.3 ± 0.2
*C*_max_ (μM)	0.2 ± 0.1	0.1 ± 0	2.1 ± 0.6	0.8 ± 0.1	2.0 ± 0.1	1.4 ± 0.3
*t*_max_ (min)	30 ± 0	83 ± 70	45 ± 15	45 ± 25	45 ± 15	186 ± 64
AUC_0-*t*_ (μM ⋅ h)	0.6 ± 0.2	0.4 ± 0.1	5.1 ± 0.2	3.0 ± 0.4	6.3 ± 0.3	11.9 ± 3.0
AUC_0-*t*_/dose [μM ⋅ h/(mg/kg)]	0.4 ± 0.1	0.2 ± 0	0.5 ± 0.1	0.3 ± 0.1	0.3 ± 0	0.5 ± 0.2
*F* (%)	50 ± 11	32 ± 5	77 ± 3	45 ± 6	44 ± 1	89 ± 24

Values shown are means ± sd (*n* = 3). N.d., not determined.

#### *In vivo* β_1_- vs. β_2_-selectivity—intravenous studies

**Figure 8.**
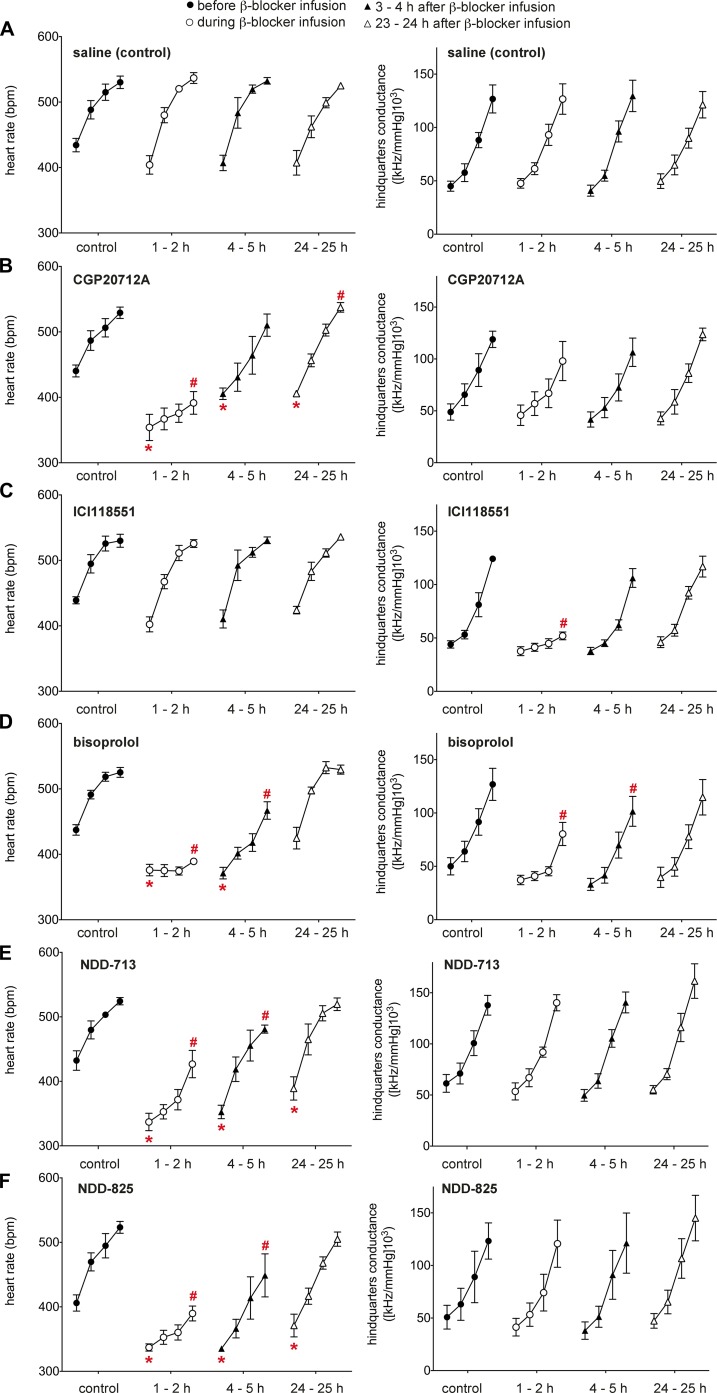
Selectivity of various β-blockers *in vivo*. Responses to isoprenaline before and after administration of intravenous saline or β-blockers in conscious, atropine-treated, freely moving rats. Absolute values for HR and HQC were measured before (first value of each linked series) and at the end of 3-min infusions of isoprenaline (12, 40, and 120 ng/kg/min; remaining 3 values in each linked series). Isoprenaline was administered before (control, solid circles), during (open circles), 3–4 h after (solid triangles), and 23–24 h after (open triangles) the intravenous infusion of saline (0.1 ml/kg bolus; 0.4 ml/kg/h infusion; *A*), CGP20712A (200 μg/kg bolus; 100 μg/kg/h infusion; *B*), ICI118551 (2 mg/kg bolus; 1 mg/kg/h infusion; *C*), and bisoprolol (2 mg/kg bolus; 1 mg/kg/h infusion; *D*), NDD-713.HCl (2 mg/kg bolus: 1 mg/kg/h infusion; *E*), and NDD-825.HCl (2 mg/kg bolus; 1 mg/kg/h infusion; *F*). Values are means ± sem (*n* = 4). *P < 0.05 *vs*. baseline before β-blocker; ^#^*P* < 0.05 *vs*. isoprenaline maximum value before β-blocker (Quade test).

To assess β_1_-selectivity *in vivo*, compounds were administered to conscious rats, and HR (β_1_) and HQC (β_2_) were monitored (51). Infusion of increasing concentrations of the nonselective β-AR agonist isoprenaline resulted in dose-dependent increases of HR and HQC (**Fig. 8**). Infusion of CGP20712A suppressed both basal and isoprenaline-stimulated HR, with no significant reduction of HQC, whereas ICI118551 suppressed a isoprenaline-induced increase in HQC but not HR (Fig. 8). Bisoprolol (moderate β_1_-selectivity at rat β-ARs and low β_1_-selectivity at human β-ARs; Table 1) reduced basal and isoprenaline-induced HR responses but also suppressed the HQC response. NDD-713 and -825 suppressed basal and isoprenaline-induced HR but had no effect on HQC responses, which confirmed their high β_1_-selectivity. These effects were still visible at 4.0–5.5 h (3 h after i.v. infusion finished) and suppression of basal activity was also observed at 24–25 h (23–24 h after intravenous infusion finished), which was in keeping with the longer PK clearance of NDD-713 and -825.

#### *In vivo* β_1_- vs. β_2_-selectivity—oral studies

**Figure 9.**
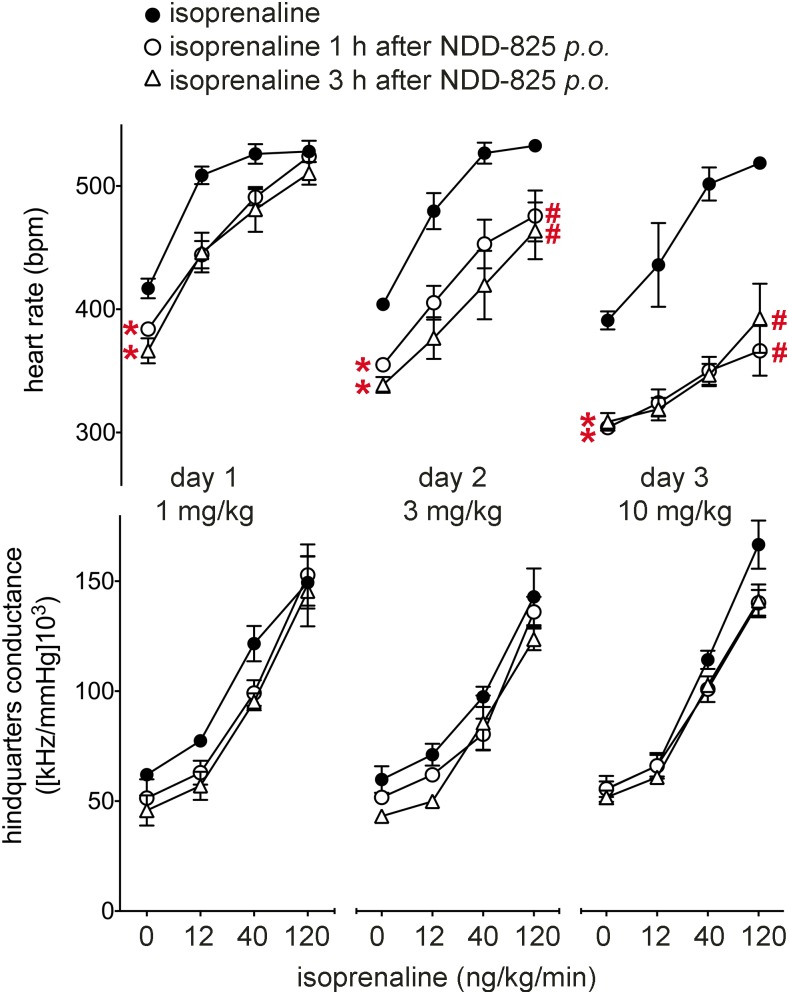
Responses to isoprenaline before and after administration of β-blockers in conscious, atropine-treated, freely moving rats. Absolute values for HR and HQC were measured before (first value of each linked series) and at the end of 3-min infusions of isoprenaline (12, 40, and 120 ng/kg/min; remaining 3 values in each linked series). Isoprenaline was administered before (control, solid circles), 1 h (open circles), and 3 h (open triangles) after NDD-825.HCl, p.o. at a dose of 1 mg/kg (d 1), 3 mg/kg (d 2), and 10 mg/kg (d 3). Values are means ± sem (*n* = 3 rats). *P < 0.05 *vs*. baseline before β-blocker; ^#^*P* < 0.05 *vs*. isoprenaline maximum value before β-blocker (Quade test).

NDD-825 was administered by oral gavage (**Fig. 9**). At 1 mg/kg, NDD-825 caused a small but significant lowering of basal HR. This was more pronounced on subsequent days (at doses of 3 and 10 mg/kg), as was the reduction in isoprenaline-stimulated HR. No significant changes in HQC responses to isoprenaline were recorded at any dose level.

#### Safety pharmacology and 7-d toxicology study

NDD-713 and -825 showed no cytotoxic effects in HepG2 cell viability assays even at the limits of aqueous solubility. They had no affinity for the hERG channel (at concentrations >3000-fold β_1_-AR affinity) and only blocked the hERG potassium current at high concentrations (IC_50_ > 10 μM). Neither compound displayed genotoxicity at concentrations of ≤10 μM in Ames tests. In the 7-d repeat-dose oral rat toxicology study, NDD-825 was well tolerated at the highest dose tested (300 mg/kg once daily) without any macro- or microscopic clinical changes at autopsy. The most significant change was a dose-related increase in triglycerides at the top dose, which is a well-recognized effect of β-blockers (58); however, the maximum tolerated dose was not achieved and is therefore >300 mg/kg daily.

## DISCUSSION

β-Blockers are important for reducing mortality in cardiovascular disease. Although clinical studies have suggested that β-blockers are associated with a reduction in lung function in the general population (14), β-blockers are well tolerated by most people. However, current clinical β-blockers are poorly β_1_-selective and risk bronchospasm and the loss of effectiveness of β_2_-agonist rescue (13), and, thus, are contraindicated in patients with asthma (25). This poor selectivity also underlies safety concerns about β-blockers in two other cardiovascular high-risk groups: patients with COPD (bronchospasm) and those peripheral vascular disease (β_2_-mediated vasoconstriction).

CGP20712A and LK204-545 are highly β_1_-selective β-blockers, but neither were developed into clinical drugs. CGP20712A interacts with many other receptors (Fig. 6), and LK204-545 has significant partial agonism, which increases HR in rats (42). As there are currently no highly β_1_-selective ligands without these problematic properties, we aimed to develop these novel ligands.

NDD-713 and -825 had high affinity for human β_1_-AR and low affinity for human β_2_- and β_3_-ARs in two direct measures of affinity, which gave high β_1_-selectivity compared with clinical β-blockers. NDD-713 and -825 also had longer durations of action at β_1_-AR than did bisoprolol, although these were shorter than those of nebivolol and carvedilol. CGP20712A and LK204-545 bound to a significant number of other targets, whereas NDD-713 and -825 had poor affinity for most other GPCRs and ion channels. The highest off-target affinity was for the 5-HT2A GPCR for which β_1_-selectivity over the 5-HT2A receptor was 510-fold for NDD-713 and 1115-fold for NDD-825. Unlike NDD-825, -713 also had some affinity for the noradrenaline transporter (β_1_-AR *vs.* noradrenaline transporter selectivity was 185-fold) and the dopamine transporter (β_1_-AR *vs*. dopamine transporter selectivity was 53-fold); therefore, NDD-825 has a clean off-target profile.

Having established high β_1_-selectivity *in vitro*, we investigated *in vivo* selectivity by using the conscious, freely moving rat model that was previously used to distinguish β_1_- and β_2_-selective compounds (51). Here, HR responses are purely β_1_ mediated, whereas HQC responses are purely β_2_ mediated. Intravenous NDD-713 and -825 both reduced basal and isoprenaline-stimulated HR but did not reduce HQC (Fig. 8), which suggested no blockade of rat vascular β_2_-ARs. As this may be considered a surrogate marker for β_2_-AR interaction in airways, NDD-713 and -825 affinity was also determined in human bronchial smooth muscle cells, the main target of β_2_-agonist therapy in asthma and COPD. Here, NDD-713 and -825 affinity for native human β_2_-AR was poor. Overall, these studies confirmed the highly β_1_-selective natures of NDD-713 and -825.

Previous studies have shown that the degree of partial agonism observed in CHO-β_1_ cells directly reflects the amount of β_1_-mediated partial agonism (increase in HR) observed in rats (51). Thus, xamoterol and bucindolol, with substantial β_1_-mediated partial agonism, stimulate HR, but carvedilol (lower partial agonism in CHO-β_1_ cells) and bisoprolol (no partial agonism) reduce HR (51). Of the 4 β-blockers that similarly reduce mortality in heart failure (7), carvedilol has the highest degree of partial agonism, and bisoprolol and metoprolol have no agonism or bias agonism at all. Thus, intrinsic activity that is low, equal to, or (ideally) below that of carvedilol is required, but the presence or absence of bias is not important. In CHO-β_1_ cells, NDD-713 and -825 did not stimulate any agonist response at cAMP, CRE-gene transcription (a sensitive readout of both cAMP and ERK1/2 MAPK), or by directly measuring ERK1/2 MAPK, which suggests no agonism or biased agonism. In rats, NDD-713 and -825 reduced both basal and isoprenaline-stimulated HR, which once again confirmed no partial agonism (ISA). Both *in vitro* and *in vivo* studies, therefore, demonstrate that NDD-825 and -713 are neutral antagonists and, thus, have an efficacy profile similar to that of bisoprolol, metoprolol, and CGP20712A, rather than carvedilol.

To assess pharmacologic activity by the oral route, NDD-713, -825 and bisoprolol were administered by oral gavage to lightly sedated rats and measurements taken once rats were fully recovered. All 3 compounds caused a reduction of basal and isoprenaline-stimulated HR, which confirmed good oral bioavailability. For NDD-825, where multiple different doses were administered, the reduction in basal and isoprenaline-stimulated HR was dose dependent, and a reduction in basal HR was observed the following day, which suggested a long duration of action.

This longer duration of action (compared with bisoprolol) was also observed in rat PK studies. After 20 mg/kg, p.o., plasma *f*_u_ level of bisoprolol exceeded the β_2_-AR *K*_d_ value for >5 h (Fig. 7). Bisoprolol-mediated β_2_-AR blockade would likely be manifest for 5 h and the β_1_-AR–mediated effects would probably last for ∼8 h. PK analysis of NDD-713 and -825 (20 mg/kg p.o.; Tables 5 and 6) revealed slow oral absorption and elimination and large volumes of distribution; therefore, they are likely to block β_1_-AR for much longer periods (*i.e.,* compare 10 h for NDD-713 and >24 h for NDD-825). Of importance, unbound plasma level for both compounds never reached the respective β_2_-AR *K*_d_ values at any time, and even at *C*_max_, were at least 5-fold below β_2_-AR *K*_d_, which suggests that β_2_-AR blockade by NDD-713 or -825 is unlikely.

Drug metabolism and PK properties suggest that NDD-713 and -825 have excellent characteristics that lead to extensive exposure at comparatively low doses, similar to those of bisoprolol but with significantly slower clearance and elimination. In addition, no safety problems were identified, with no cytotoxic, genotoxic, or hERG binding identified, and no problematic clinical chemistry, hematologic, microscopic, or macroscopy organ changes identified (7-d dosing, up to 300 mg/kg/d). NDD-713 and -825, therefore, seem to be safe to administer to rats, even at high doses.

Finally, to understand why such a high degree of β_1_- *vs.* β_2_-selectivity is required to discriminate effects on cardiovascular and respiratory function, we calculated the SIs of NDD-713 and NDD-825 and compared these with those of bisoprolol. NDD-713 and NDD-825 have high β_1_-AR binding selectivity at the receptor level (SI_1_; **Table 7**). We assessed the relative *in vivo* receptor occupancies (Φ) of heart β_1_-AR and lung β_2_-AR as a function of unbound drug levels in plasma for bisoprolol. Bisoprolol plasma concentrations, measured in humans after 10 mg/d bisoprolol, p.o., were obtained from published data (59, 60) to yield an SI (SI_2_; Table 7) that takes into account relative receptor occupancies and *f*_u_ of drug in plasma at steady state (*C*_ssf_). For bisoprolol, with 43- to 69-fold β_1_-AR selectivity at the receptor level (SI_1_), the apparent β_1_-AR selectivity is reduced to 6-fold (SI_2_) when taking into account *in vivo* receptor occupancy.

**TABLE 7.** Comparison SIs in terms or receptor binding (SI_1_), receptor occupancy (SI_2_), and pharmacological response (SI_3_) for bisoprolol, NDD-713, and -825

β-Blocker	SI_1a_ (*in vitro* binding)*^a^*	SI_1b_ (*in vitro* inhibition of functional response)*^b^*	SI_2_ (*in vivo r*eceptor occupancy)*^c^*	SI_3_ (*in vivo* pharmacologic response)*^d^*
NDD-713	589	1202	67	67
NDD-825	1023	1738	115	81
Bisoprolol	43	69	6	4

a SI_1a_ = *K*_d,β2_/*K*_d,β1_ (*K*_d_ values from ^3^H-CGP12177 whole-cell binding; Table 1). *^b^*SI_1b_ = *K*_d,β2_/*K*_d,β1_ (*K*_d_ values from antagonism of cimaterol-induced ^3^H-cAMP accumulation; Table 2). *^c^*SI_2_ = Φ_β1_/Φ_β2_ = (*K*_d,β2_ + *C*_ssf_)/(*K*_d,β1_ + *C*_ssf_), where Φ is the receptor occupancy at the βARs, and *C*_ssf_ values refer to unbound drug levels in plasma at steady state upon once-daily oral dosing (*K*_d_ values from Table 1). *^d^*SI_3_ = *E*_β1_/*E*_β2_, where *E*_β1_ and *E*_β2_ refer to effects on exercise HR and on FEV1 under steady-state conditions after drug administration, respectively.

A PK–pharmacodynamic model (23, 54) was then applied that considers relative β-AR affinity, receptor occupancy, and relative receptor numbers in target organs (61) and how receptor responses are actually transduced to the pharmacologic responses [*i.e.,* reduction of exercise-induced HR for β_1_-AR (62) and reduction in lung function (FEV1) for β_2_-AR (63)]. We calculated a third SI (SI_3_; Table 7) that expresses selectivity in terms of *in vivo* pharmacologic responses (*E*) according to a ternary complex model (23, 54). We thus determined an SI_3_ value of 4 for bisoprolol. It is clear that with such a low *in vivo* pharmacologic selectivity, bisoprolol could cause bronchospasm in susceptible individuals.

We used the known ratio between bisoprolol exposure (*C*_ssf_) at an efficacious dose (10 mg/d, p.o., which lowers exercise-induced HR in humans) and receptor affinity (*K*_dβ1-AR_) to estimate SIs for NDD-713 and NDD-825 (Table 7). Much higher indices (67- to 81-fold) were obtained for NDD compounds, which suggests greater *in vivo* selectivity than bisoprolol. The results demonstrate that to obtain significant separation of *in vivo* pharmacologic responses with respect to cardiac and respiratory function, high selectivity with regard to binding to β_1_-AR *vs.* β_2_-AR is required.

## CONCLUSIONS

NDD-713 and, in particular, NDD-825 are high-affinity, β_1_-selective ligands that are devoid of ISA, off-target effects, and toxicology issues, but with good PK–pharmacodynamic and absorption, distribution, metabolism and elimination properties that maintain β_1_-selectivity in conscious animals. These ligands are therefore promising candidates for the development of β-blockers devoid of β_2_-AR–mediated adverse effects of bronchospasm and vasoconstriction and, thus, may prove beneficial in patients with concomitant cardiovascular and respiratory disease or limb ischemia (peripheral vascular disease).

## Abbreviations

AR: adrenoceptor
CHO: Chinese hamster ovary
CL_int_: intrinsic clearance
COPD: chronic obstructive pulmonary disease
CRE: cAMP response element
FEV1: forced expiratory volume in 1 s
*f*_u_: unbound fraction
HQC: hindquarters vascular conductance
HR: heart rate
ISA: intrinsic sympathomimetic activity
LC-MS: liquid chromatography–mass spectrometry
P450: cytochrome P450
PK: pharmacokinetic
SI: selectivity index
SPAP: secreted alkaline phosphatase
UPLC-MS: ultraperformance liquid chromatography–mass spectrometry
